# Akt activity protects rheumatoid synovial fibroblasts from Fas-induced apoptosis by inhibition of Bid cleavage

**DOI:** 10.1186/ar2941

**Published:** 2010-02-26

**Authors:** Samuel García, Myriam Liz, Juan J Gómez-Reino, Carmen Conde

**Affiliations:** 1 Research Laboratory and Rheumatology Unit, Hospital Clínico Universitario, Choupana s/n, Santiago de Compostela, 15706-Spain; 2 Department of Medicine. University of Santiago de Compostela. San Francisco s/n, Santiago de Compostela, 15782-Spain

## Abstract

**Introduction:**

Synovial hyperplasia is a main feature of rheumatoid arthritis pathology that leads to cartilage and bone damage in the inflamed joints. Impaired apoptosis of resident synoviocytes is pivotal in this process. Apoptosis resistance seems to involve defects in the extrinsic and intrinsic apoptotic pathways. The aim of this study was to investigate the association of PI3Kinase/Akt and the mitochondrial apoptotic pathway in the resistance of rheumatoid arthritis (RA) fibroblast like synovial cells (FLS) to Fas-mediated apoptosis.

**Methods:**

Apoptosis was assessed by ELISA quantification of nucleosomal release, Hoechst staining and activated caspase-3/7 measure in cultured RA FLS stimulated with anti-Fas antibody. Two Phosphoinositol-3-kinase/protein Kinase B (PI3 Kinase) inhibitors, Wortmannine and LY294002, were used before anti-Fas stimulation. Proapoptotic BH3 interacting domain death agonist (Bid) was suppressed in RA FLS by small interfering RNA (siRNA) transfection. Bid was overexpressed by transfection with the pDsRed2-Bid vector. Phosphorylated Akt, caspase-9, and Bid expression were analysed by western blot.

**Results:**

PI3 kinase inhibition sensitizes RA FLS to Fas-induced apoptosis by increasing cleavage of Bid protein. Bid suppression completely abrogated Fas-induced apoptosis and Bid overexpression highly increased apoptotic rate of RA FLS in association with cleavage of caspase-9.

**Conclusions:**

In RA FLS, phosphorylation of Akt protects against Fas-induced apoptosis through inhibition of Bid cleavage. The connection between the extrinsic and the intrinsic apoptotic pathways are critical in this Fas- mediated apoptosis and points to PI3Kinase as potential therapeutic target for RA.

## Introduction

In rheumatoid arthritis (RA) joints synovial hyperplasia and inflammatory cell infiltration lead to progressive destruction of cartilage and bone. Although the mechanisms underlying synovial hyperplasia are not completely known, accumulating evidence suggests that alterations in the apoptosis of synoviocytes are pivotal [1-3].

Interestingly, RA fibroblast-like synoviocytes (FLS) express death receptors; yet, they are relatively resistant to FasL, TNF, and tumor necrosis (TNF)-related apoptosis-inducing ligand (TRAIL)-induced apoptosis [3-5]. This resistance has been related to high expression of anti-apoptotic molecules such as Fas-associated death domain-like IL1 beta-converting enzyme-inhibitory protein (FLIP) [6,7], sentrin-1 [8,9], Bcl-2 [10], Mcl-1 [11], and constitutive activation of Akt [12-14].

Apoptosis is a process highly regulated and crucial in many physiological situations, and could involve two main pathways; the extrinsic, by activation of death receptors (Fas, TNF-RI), and the intrinsic or mitochondrial pathway. In the extrinsic pathway, FasL, TNF, and TRAIL ligation leads to recruitment of Fas-associated via death domain (FADD) and procaspase-8, which form the death inducing signaling complex (DISC), where caspase-8 is activated. In turn, caspase-8 activates caspase-3, which causes DNA fragmentation and cell death. The mitochondrial pathway is induced by hypoxia, cytotoxic drugs and growth factor deprivation leading to liberation of cytochrome c (cyt c) and Apaf-1-mediated activation of the caspase-9 [15-17]. This pathway is tightly regulated by members of the Bcl-2 family with anti-apoptotic function, such as Bcl-2, Bcl-xL, Bcl-w, Mcl-1, and A1, which prevent mitochondrial membrane permeability and release of cyt c. In contrast, other Bcl-family members, such as Bax, Bak, Bok, BH3 interacting domain death agonist (Bid), Bad, Bim, and Puma, are pro-apoptotic and promote mitochondrial membrane permeability [18]. In some cell types, named type II cells, the two apoptotic pathways are connected through the cleavage of Bid by activated caspase-8. Truncated Bid translocates to the mitochondria causing release of cyt c and cell death [19]. In contrast, in type I cells, death-receptor induced apoptosis is independent of Bid [19]. It seems that both the intrinsic and extrinsic apoptotic pathways are involved in arthritis development. There is much evidence implicating the extrinsic pathway [review in ref [20] and [21]]. However, support for the role of the intrinsic pathway is scant, although very convincing. For example, mice lacking Bim [22] or Bid [23] develop a severe synovial inflammation and bone destruction in an arthritis model. Also, evidence suggests that RA FLS are type II cells [24]. Therefore, it is necessary to investigate the relevance of the intrinsic pathway and its connection with the extrinsic pathway in the FLS resistance to apoptosis.

RA FLS typically show Akt activation that could contribute to the relative resistance to apoptosis by unknown mechanisms. Akt/PKB is a Ser/Thr protein kinase implicated in inhibition of apoptosis and stimulation of cellular growth in several tissues by mechanisms including phosphorylation of the pro-apoptotic proteins Bad [25] and Bax [26], and suppression of pro-apoptotic proteins such as Bim and PUMA, through phosphorylation of the forkhead pathway [27]; favouring the anti-apoptotic effect of Mdm2 on p53 [28]; and inhibition of cleavage of Bid protein [29,30].

The aim of this study was to investigate the connection of the death receptor stimulation with the intrinsic pathway in the apoptosis of the type II cells RA FLS, and to analyse the possible relation between constitutively activated phosphoinositol-3 (PI3) kinase/Akt and the mechanisms of resistance to Fas-mediated apoptosis.

## Materials and methods

### Fibroblast-like synoviocytes

FLS from 11 patients with RA were obtained at the time of synovectomy or total joint replacement. All RA patients fulfilled the American College of Rheumatology 1997 criteria for RA classification [31]. All patients gave informed, written consent. The study was performed according to the recommendations of the Declaration of Helsinki and with the approval of the Comité Etico de Investigación Clínica de Galicia. Synovial tissue was minced and incubated with 10 μg/ml collagenase in serum-free DMEM (Gibco Invitrogen, Barcelona, Spain) for three hours at 37°C. After digestion, FLS were filtered through a nylon cell strainer (BD Falcon, Franklin Lakes, NJ 07417 USA), washed extensively with DMEM, and cultured in DMEM supplemented with 10% v/v FCS (Gibco Invitrogen, Barcelona, Spain), 1% penicillin-streptomycin (Gibco Invitrogen, Barcelona, Spain) and 1% L-glutamine (Sigma, St Louis, MO, USA) in a humidified 5% carbon dioxide atmosphere. Adherent cells were trypsinized and splited in a 1:3 ratio once the cells were 80 to 90% confluent. FLS from passages three to eight were used.

### Small interfering RNA transfection in FLS

Bid small interfering RNA (siRNA), a pool of four target-specific 19 nucleotide siRNAs, and non-silence control siRNA, a pool of four non-targeting siRNAs, were purchased from Dharmacon (Fisher Bioblock Scientific, Strasbourg, France). siRNA transfections were performed as described elsewhere [32]. Briefly, RA FLS at 80 to 90% confluence were transiently transfected with siRNA (200 nM) in Opti-MEM I (Gibco Invitrogen, Barcelona, Spain) using 1.25 μg/ml DharmaFECT 1 (Dharmacon, Fisher Bioblock Scientific, Strasbourg, France). Bid suppression was analysed by western blot. Experiments were performed 48 hours after transfections.

### pDsRed2-Bid Vector transfection in FLS

pDsRed2-Bid Vector, a 5.3 Kb mammalian expression vector that encodes a fusion of Discosoma sp red fluorescent protein (DsRed2) and Bid, and the empty pDsRed2 vector, were purchased from Clontech (Mountain View, CA, USA). RA FLS at 60% confluence were transiently transfected with 0.5 μg pDsRed2-Bid vector or pDsRed2 vector in Opti-MEM I (Gibco Invitrogen, Barcelona, Spain) using 4 μg/ml Lipofectamine (Gibco Invitrogen, Barcelona, Spain) and 9 μg/ml Plus Reagent (Gibco Invitrogen, Barcelona, Spain). Bid expression was analysed by western blot and immunofluorescence assays. Experiments were performed 48 hours after transfections.

### Apoptosis and cell death assays

RA FLS (3 × 10^3^) were cultured in 96-well plates with DMEM and 5% FCS. Forty-eight hours after transfection, cells were treated for one hour with 10 μM LY294002 (LY), 1 μM wortmannin (Wort; Sigma, St Louis, MO, USA) or 10 μM Z-LE(OMe)HD(OMe)-FMK (Calbiochem-Merck KGaA, Darmstadt, Germany) and then incubated for 12 hours either with 1 μg/ml of human anti-Fas, clone 11 (Cell Signaling, Beverly, MA, USA) or with 100 ng/ml of membrane bound Fas ligand (memFasL, Millipore, Molsheim, France), when indicated. Apoptosis was determined by quantifying mono- and oligonucleosomal DNA using the Cell Death Detection ELISA kit (Roche Diagnostics, Indianapolis, IN, USA) as previously described [14]. Apoptosis was confirmed by Hoechst staining and measure of activated caspase-3/7 by the Caspase-Glo 3/7 assay (Promega Biotech Ibérica, S.L., Spain). RA FLS (10^4^) were cultured either on 24-well plates (Hoechst assay) or 96-well plates (Caspase-Glo 3/7 assay), treated for one hour with 1 μM Wort or 10 μM LY and then incubated for 12 hours with 1 μg/ml of human anti-Fas. After incubation, plates were stained with 10 μg/ml Hoechst 33258 (Sigma, St Louis, MO, USA), fixed with 4% paraformaldehyde and the cells were examined by fluorescence microscopy. For activated caspase-3/7 analysis, cells were incubated for one hour with reconstituted Caspase 3/7-Glo reagent and then, the luminescence signal generated after cleavage of DEVD-aminoluciferin substrate by caspase 3/7, was measured using a Fluostar OPTIMA microplate reader (BMG Labtech, Offenburg, Germany).

### Western blot analysis

After siRNA transfections, RA FLS (8.5 × 10^4^) were cultured in six-well plates, treated for one hour with 1 μM Wort and then stimulated with human anti-Fas 1 μg/ml for 3 or 12 hours. Cells were washed twice with ice-cold PBS, and protein was extracted using lysis buffer (50 mM Tris HCl [pH 7.5], 250 mM NaCl, 1% Triton X-100, 30 mM NaPO, 5 mM EDTA (pH 8.0), 100 mM NaF, 1 mM Na_3_VO_4_, 10 μg/ml aprotinin, 10 μg/ml leupeptin, and 1 mM phenylmethylsulfonyl fluoride). The protein concentrations in the extracts were determined using the Qubit fluorometer (Invitrogen, C/Garrotxa 10-12, Prat de Llobregat, Barcelona, Spain) according to the manufacturer's protocol. Whole cell lysates (40 to 50 μg of protein) were fractionated by Tris-glycine buffered 10% SDS-PAGE and transferred to polyvinylidene fluoride (PVDF) membrane (Hybond-P, Amersham Biosciences, Little Chalfont, UK). The membranes were blocked with Tris buffered saline and 0.1% Tween-20 (TBST) containing 5% non-fat milk for two hours at room temperature, followed by incubation with antibody to phospho-Akt, Akt, Bid, Caspase-9 (Cell Signalling, Beverly, MA, USA) or β-actin (Sigma, St Louis, MO, USA) overnight at 4°C. After washing with TBST, the membrane was incubated with horseradish peroxidase-conjugated secondary antibody (Santa Cruz, Bergheimer Str 89-2, Heidelberg, Germany for one hour at room temperature. Immunoreactive protein was detected with Immobilion western (Milipore, MA, USA).

### Statistical analysis

Differences between experimental groups were assessed by Wilcoxon matched-pairs test. *P *values less than 0.05 were considered significant.

## Results

### Regulation of Fas-mediated apoptosis in RA FLS by Akt

RA FLS from six patients were pre-treated for one hour with Wort or LY, and stimulated thereafter with Fas-antibody for 12 hours. Apoptosis of RA FLS was determined by analysis of nucleosomal release, Hoechst staining and activated caspase-3/7 measurement. As a positive control we analysed the nucleosomal release after anti-Fas stimulation in Jurkat cells. Mean DO492 nm was 0.93 versus a mean of 0.13 observed in the six RA FLS, confirming the relative resistance of these latter cells to Fas-induced apoptosis.

**Figure 1:**
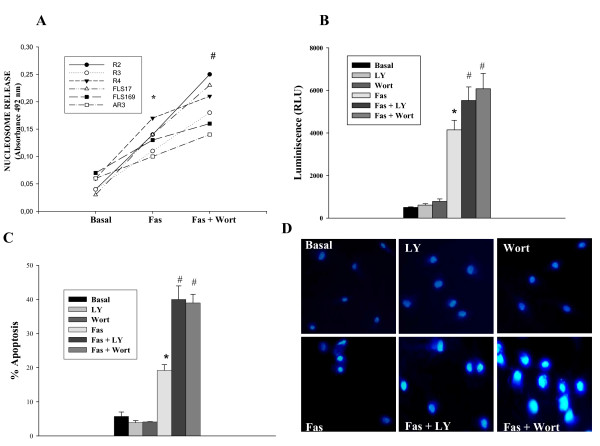
**PI3 kinase inhibition sensitizes RA FLS to Fas-induced apoptosis**. Cultured rheumatoid arthritis (RA) fibroblast-like synoviocytes (FLS) were untreated (Basal), treated for 12 hours with 1 μg/ml anti-Fas antibody or pretreated for one hour with phosphoinositol-3 (PI3) kinase inhibitors (Wortmannine (Wort) or LY294002 (LY)) before Fas stimulation. **(a) **Apopotosis was quantified by nucleosomal release ELISA assay. Changes in individual RA FLS lines are represented. * indicates *P *< 0.05 versus untreated cells; # indicates *P *< 0.01 versus anti-Fas-only treated cells. **(b) **Caspase 3/7 activity determined by a caspase 3/7-Glo assay. **(c) **Quantification of apoptosis in RA FLS by Hoetchst 33258 staining. For b and c, results are the mean (standard error of the mean) of six different RA FLS lines. * indicates *P *< 0.05 versus untreated cells and # indicates *P *< 0.05 versus anti-Fas-only treated cells. **(d) **Representative images of one experiment are shown.

In RA FLS, anti-Fas stimulation induced significant apoptosis compared with the basal situation (Figures 1a to 1d). Treatment with Wort or LY did not induce cell death by themselves, whereas when combined with anti-Fas they significantly increased the apoptotic rate when compared with anti-Fas alone, as has been shown in our previous work [14] (Figures 1a to 1d).

### Connection between the intrinsic and extrinsic apoptotic pathways in RA FLS

**Figure 2:**
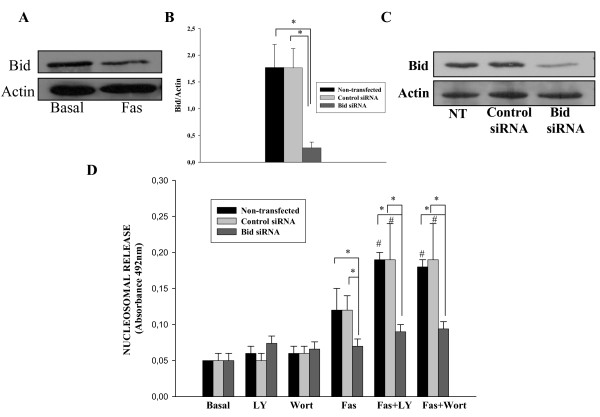
**Effect of Bid suppression on Fas-induced apoptosis of RA FLS**. **(a) **Cultured fibroblast-like synoviocytes (FLS) from five rheumatoid arthritis (RA) patients were incubated with 1 μg/ml of anti-Fas antibody and Bid expression was assessed by western blot. A representative blot is shown. **(b and c) **RA FLS were either non-transfected or transfected with Bid or control small interfering RNA (siRNA). Proteins were extracted 48 hours after transfection and western blot was performed. **(b) **Densitometric analysis of band intensities normalized to the actin band is shown. Data are the mean (standard error of the mean (SEM)) of five different RA FLS lines. * indicates *P *< 0.05. **(c) **A representative blot is shown. **(d) **Forty-eight hours after transfection, RA FLS were left untreated (Basal), treated for 12 hours with 1 μg/ml anti-Fas antibody or pretreated for one hour with Wortmannine (Wort) or LY294002 (LY) before Fas stimulation. Apoptosis was determined by quantification of nucleosomal release. Results are the mean (SEM) of six different RA FLS lines. * indicates *P *< 0.05 versus basal; and # indicates *P *< 0.05 versus anti-Fas-only treatment.

There is some indication that RA FLS are type II cells in relation to apoptosis because Bid was cleaved after anti-Fas stimulation [24]. We have confirmed these results showing that after incubation with anti-Fas the detectable full Bid protein is significantly decreased in all RA FLS lines analysed (Figure 2a). Furthermore, we wanted to know whether the cleavage of Bid is essential for apoptosis in RA FLS. To this end, Bid was suppressed in RA FLS from five different patients and the efficiency of Bid silencing is shown in Figures 2b and 2c. Interestingly, suppression of Bid completely abrogated Fas-induced apoptosis (Figure 2d). In contrast, transfection with control siRNA did not alter Fas-induced apoptosis, indicating the relevance of the Bid protein in apoptosis induced by anti-Fas, and consequently the connection between intrinsic and extrinsic pathways.

### Regulation of Bid cleavage by the PI3K/Akt pathway

**Figure 3:**
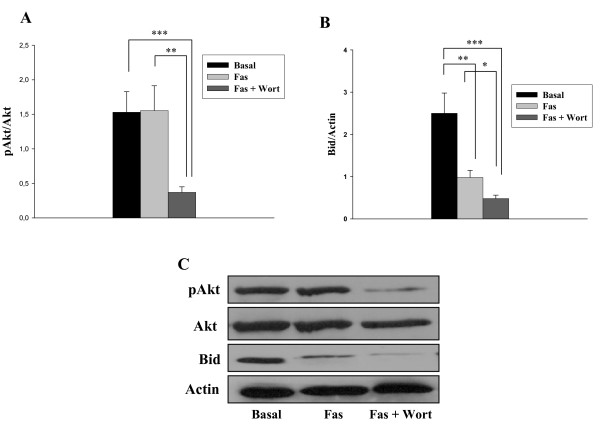
**PI3 kinase inhibition increases Bid cleavage**. Rheumatoid arthritis (RA) fibroblast-like synoviocytes (FLS) were left untreated (Basal) or treated for 12 hours with 1 μg/ml anti-Fas antibody or pretreated for one hour with phosphoinositol-3 (PI3) kinase inhibitor Wortmannine (Wort) before Fas stimulation. Expression of **(a) **phosphoAkt and **(b) **Bid was analysed by western blot. Data are expressed as mean (standard error of the mean) of FLS from six different RA patients. * indicates *P *< 0.05, ** indicates *P *< 0.01 and *** indicates *P *< 0.001. **(c) **A representative blot is shown.

Given the above results, it seemed possible that RA FLS could resemble human prostate cancer lines, in which the PI3K/Akt pathway interferes with TRAIL-mediated apoptosis by inhibiting the cleavage of Bid [29,30]. To test whether a similar mechanism was at play in RA FLS, we analysed the effect of Akt inhibition on Bid expression. For this, RA FLS from six different patients were treated with the PI3 kinase inhibitor Wort for one hour before the addition of anti-Fas antibody. As shown in Figure 3, this treatment significantly reduced the level of Akt phosphorylation and markedly increased the cleavage of Bid in comparison to that observed after anti-Fas alone. This later effect was demonstrated by a marked reduction of cellular Bid protein expression.

### Relevance of Bid cleavage for Akt contribution to Fas-induced apoptosis resistance

To further assess the contribution that regulation of Bid cleavage by Akt has on the Fas-mediated resistance to apoptosis in RA FLS, we used siRNA suppression of Bid. RA FLS non-transfected and transfected with control or Bid siRNA were pre-treated with the PI3 kinase inhibitors LY or Wort before Fas stimulation and apoptosis rate was determined (Figure 2d). Neither treatment with LY nor treatment with Wort alone induced apoptosis in RA FLS, whereas Fas stimulation after pre-treatment with any of these two inhibitors induced significant apoptosis compared with Fas-only treatment. The same result was observed in cells transfected with control siRNA, but not in cells transfected with the specific Bid siRNA, where full resistance to Fas-induced apoptosis was found both with and without Wort treatment.

### Bid availability limits Fas-induced apoptosis in RA FLS

**Figure 4:**
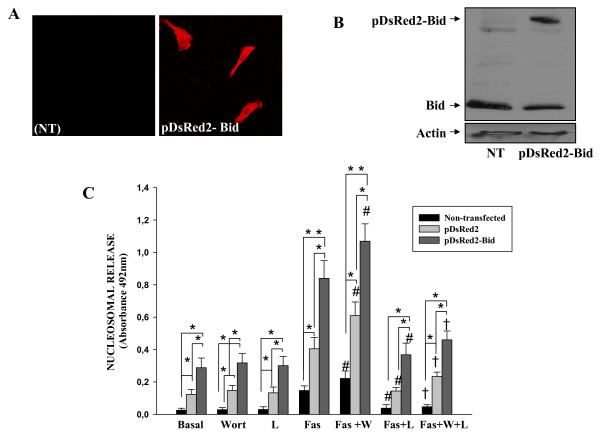
**Effect of Bid overexpression on anti-Fas induced apoptosis of RA FLS**. Rheumatoid arthritis (RA) fibroblast-like synoviocytes (FLS) were transfected with pDsRed2-Bid or pDsRed2 control vector, 48 hours after transfection Bid expression was analysed by **(a) **immunofluorescence and **(b) **western blot. Representative experiments are shown. **(c) **After transfection, cells were left untreated (Basal) or treated for 12 hours with 1 μg/ml anti-Fas antibody or pretreated for one hour with Wortmannine (Wort) or with Wort and the caspase-9 inhibitor, Z-LE(OMe) HD (O Me) FMK (LEHD), before Fas stimulation. Apoptosis was analysed by ELISA. Data are the mean (standard error of the mean) of six RA FLS lines. * indicates *P *< 0.05 versus basal, # indicates *P *< 0.05 versus anti-Fas-only treatment and † indicates *P *< 0.05 versus anti-Fas and Wort treatment.

The high cleavage of Bid shown after blocking Akt phosphorylation was accompanied by a modest increase in Fas-induced apoptosis. We wondered whether availability of Bid could limit the extent of apoptosis in a way reminiscent of the resistance mediated by increased expression of anti-apoptotic molecules [6-11]. To test this possibility, cells from six different patients were transiently transfected with full-length Bid vector (pDs Red2-Bid) or pDsRed2 control vector. The efficiency of transfection was analysed by immunofluorescence assays and western blot as shown in Figures 4a and 4b. As observed in Figure 4c, the treatment with Wort alone did not alter cell viability. Interestingly, Bid overexpression highly increased Fas-induced apoptosis compared with cells transfected with pDs2Red2 control vector (*P *= 0.01, Figure 4c), indicating that the amount of Bid contributed to resistance to apoptosis. Pre-treatment with Wort further sensitizes to apoptosis the Bid-overexpressing FLS cells, indicating that in spite of the high levels of Bid, they were still regulated by phosphorylated Akt.

Finally, to test whether the mitochondrial pathway is the only one involved in these effects, we used the caspase-9 inhibitor, Z-LE(Ome)HD(Ome)-FMK (LEHD) before Fas stimulation. Treatment of cells with LEHD alone had no effect on cell viability. However, as shown in Figure 4c, caspase-9 inhibition completely blocked apoptosis induced by treatment with anti-Fas and Wort even in Bid transfected cells. This was shown by the apoptotic rate that decreased near to basal levels in all RA FLS groups.

**Figure 5:**
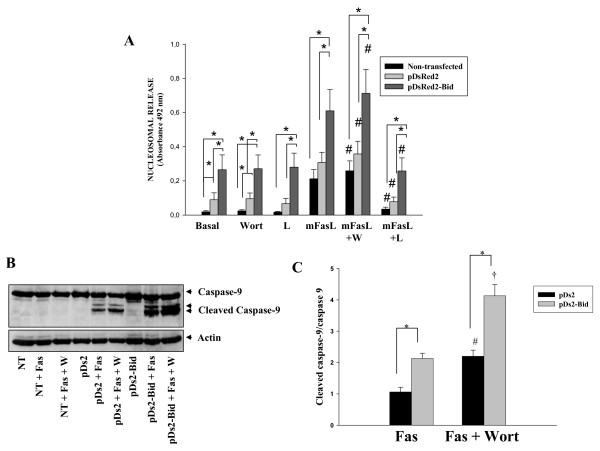
**Bid overexpression highly increases the apoptosis induced in RA FLS by membrane-bound FasL and caspase-9 cleavage after anti-Fas stimulation**. **(a) **Cultured rheumatoid arthritis (RA) fibroblast-like synoviocytes (FLS) were transfected as indicated in Figure 4 and treated for 12 hours with 100 ng/ml mFasL or pretreated for one hour with Wortmannine (Wort) or with Wort and the caspase-9 inhibitor, Z-LE(OMe) HD (O Me) FMK (LEHD), before Fas stimulation. Apoptosis, assessed by quantification of nucleosomal release, is shown and data represented the mean (standard error of the mean) of seven RA FLS different lines. **(b and c) **Transfected RA FLS were treated for 12 hours with 1 μg/ml anti-Fas antibody or pretreated for one hour with Wort before Fas stimulation. Caspase-9 cleavage was analysed by western blot. **(b) **A representative blot is shown. **(c) **Densitometric analysis of band intensity of cleaved caspase-9 normalized to full caspase-9 is shown. * indicates *P *< 0.05; # and † indicate *P *< 0.05 versus anti-Fas-only treatment.

It has been recently described that memFasL stimulation leads to more effective apoptosis than anti-Fas antibody due to different organization of DISC, leading to more efficient caspase-8 activation [33]. Then, to exclude that the Bid requirement in Fas-mediated apoptosis of RA FLS was linked to signalling with anti-Fas antibody, apoptosis was induced by treatment with memFasL. RA FLS from seven patients were treated with 1, 10 or 100 ng/ml mFasL and the 100 ng/ml was chosen as the most efficient (not shown). As shown in Figure 5a, induction of apoptosis was similar to that obtained after treatment with anti-Fas antibody. These results confirm that Bid is a limiting factor in Fas-mediated apoptosis of RA FLS under a more physiological stimulus.

We also explored by western blot the expression of caspase-9 in Bid-overexpressing and parental RA FLS after treatment with anti-Fas or anti-Fas and Wort (Figures 5b and 5c). Our results showed that PI3 kinase inhibition promotes caspase-9 cleavage that was significantly more marked in overexpressing FLS treated with Bid, confirming the mitochondrial pathway involvement.

## Discussion

Resistance of RA FLS to Fas-mediated apoptosis is of great interest not only from a scientific point of view but also for its practical implications. The synovial hyperplasia characteristic of RA is facilitated by the resistance of FLS to apoptosis. It has been demonstrated that only a small percentage of cultured FLS undergo apoptosis after Fas stimulation despite their expression of functional Fas. Furthermore, *ex vivo *studies of RA synovial tissues show that apoptotic cells are rare, although Fas receptors in FLS and its ligand in co-localized macrophages and T cells are seen [34,35]. Therefore, to elucidate the molecular mechanisms of this resistance to apoptosis, and to clarify the steps of the Fas pathway in this specific type of cells is required. Our experiments confirm that RA FLS are type II cells, in which death receptor-induced apoptosis requires activation of the mitochondrial pathway through Bid cleavage. This has already been suggested in a previous work [24]. We have also shown that constitutive Akt phosphorylation mediates the resistance to Fas-induced apoptosis in these cells. Interestingly, the effect is mediated by inhibition of the cleavage of Bid. Further to this finding, we have demonstrated that depletion of Bid by RNA interference leads to a complete resistance to Fas-mediated apoptosis in RA FLS, indicating that apoptosis signalling through Fas occurs exclusively in a Bid-dependent way. Substantiation of the role of Bid in the Fas-induced apoptosis was obtained by transfection of RA FLS with the full-length Bid vector.

Additional evidence for the involvement of the intrinsic pathway in Fas-induced apoptosis was gathered by the experiments of inhibition of caspase-9. Direct activation of caspase-3 by caspase-8 seemed insufficient to RA FLS cell death. Therefore, our results demonstrated the connection between the intrinsic and extrinsic apoptotic pathways in Fas-mediated apoptosis in RA FLS cells. In mice, Scatizzi and colleagues recently showed the importance of Bid for arthritis [23]. In K/BxN serum transfer-induced arthritis, mice lacking Bid developed severe arthritis and joint destruction. Synovial analysis showed fewer apoptotic cells in Bid-deficient mice than in control mice [23].

In addition, our work points to the PI3 kinase/Akt pathway as a novel molecular mechanism explaining the Fas-mediated resistance in RA FLS. Previous observations in RA FLS and other cell types are alike [12-14,29,30]. In RA FLS, Zhang and colleagues [13] reported that inhibition of endogenous Akt phosphorylation sensitized RA FLS to TNF-induced apoptosis. Moreover, Miyashita and colleagues [12] showed that Akt inhibition by siRNA technology significantly increased TRAIL-mediated apoptosis in RA FLS. However, the molecular mechanism has not been investigated. Recently, Audo and colleagues [36] have shown that inhibition of PI3 kinase/Akt pathway sensitizes RA FLS to TRAIL-induced apoptosis by reduction of expression of the anti-apoptotic proteins Mcl-1, XIAP, and RIP, and increase of the cell cycle inhibitor p21. Of interest in our work is that the Akt-dependent resistance to apoptosis is due to its inhibition of Bid cleavage in RA FLS cells. Therefore, Akt links the death receptor and the mitochondrial pathways in these cells. This mechanism of resistance to apoptosis has been previously reported in prostate cancer cells [29,30]. Although it is unknown how Akt regulates Bid cleavage, it is conceivable that activated Akt could phosphorylate Bid, inhibiting its cleavage by caspase-8. Indeed, it has been demonstrated that phosphorylation of Thr59, a residue localized near to the caspase-8 cleavage site, inhibits Bid cleavage by this caspase [37].

However, Akt inhibits apoptosis through several other mechanisms including activation of nuclear factor-kB, phosphorylation of Bad, Bax, and inhibition of pro-apoptotic p53 [25,26,28,38]. It seems that different cells types have different mechanisms leading to the Akt-dependent resistance to apoptosis.

## Conclusions

Our results show, for the first time, that endogenous phosphorylation of Akt protects RA FLS against the apoptosis induced by Fas through inhibition of Bid cleavage and point to PI3 kinase/Akt pathway as potential therapeutic target in RA.

In summary, this study demonstrates the essential role of the mitochondrial pathway in Fas-mediated apoptosis of RA FLS and describes a new molecular mechanism of this apoptosis resistance.

## Abbreviations

BID: BH3 interacting domain death agonist; cyt c: cytochrome c; DISC: death inducing signaling complex; DMEM: Dulbecco's modified Eagle's medium; ELISA: enzyme-linked immunosorbent assay; FADD: Fas-associated via death domain; FCS: fetal calf serum; FLIP: Fas-associated death domain-like IL1 beta-converting enzyme-inhibitory protein; FLS: fibroblast-like synoviocytes; LY: LY294002; PBS: phosphate-buffered saline; PI3: phosphoinositol-3; RA: rheumatoid arthritis; siRNA: small interfering RNA; TNF: tumor necrosis factor; Wort: Wortmannine.

## Competing interests

The authors declare that they have no competing interests.

## Authors' contributions

SG carried out the experiments and participated in the analysis of data and in drafting the manuscript. ML obtained the fibroblast-like synoviocytes and participated in western blot analysis. GJ participated in design and coordination of the study and revision of the manuscript. CC conceived of the study, participated in its design, coordination, and interpretation of data and drafted the manuscript. All authors read and approved the final manuscript.
